# 3D‐Printed Nanocarbon Polymer Conductive Structures for Electromagnetic Interference Shielding

**DOI:** 10.1002/smtd.202401822

**Published:** 2025-03-16

**Authors:** Shidhin Mappoli, Keval K. Sonigara, Suvani Subhadarshini, Martin Pumera

**Affiliations:** ^1^ Future Energy and Innovation Laboratory Central European Institute of Technology Brno University of Technology Purkynova 123 Brno 61200 Czech Republic; ^2^ Department of Chemical and Biomolecular Engineering Yonsei University 50 Yonsei‐ro, Seodaemun‐gu Seoul 03722 Republic of Korea; ^3^ Advanced Nanorobots & Multiscale Robotics Laboratory Faculty of Electrical Engineering and Computer Science VSB – Technical University of Ostrava 17. listopadu 2172/15 Ostrava 70800 Czech Republic; ^4^ Department of Medical Research China Medical University Hospital China Medical University No. 91 Hsueh‐Shih Road Taichung Taiwan

**Keywords:** 3D printing, electrodeposition, EMI shielding, fused deposition modelling, polyaniline

## Abstract

Electromagnetic interference (EMI) significantly affects the performance and reliability of electronic devices. Although current metallic shielding materials are effective, they have drawbacks such as high density, limited flexibility, and poor corrosion resistance that limit their wider application in modern electronics. This study investigates the EMI shielding properties of 3D‐printed conductive structures made from polylactic acid (PLA) infused with 0D carbon black (CB) and 1D carbon nanotube (CNT) fillers. This study demonstrates that CNT/PLA composites exhibit superior EMI shielding effectiveness (SE), achieving 43 dB at 10 GHz, compared to 22 dB for CB/PLA structures. Further, conductive coating of polyaniline (PANI) electrodeposition onto the CNT/PLA structures improves the SE to 54.5 dB at 10 GHz. This strategy allows fine control of PANI loading and relevant tuning of SE. Additionally, the 3D‐printed PLA‐based composites offer several advantages, including lightweight construction and enhanced corrosion resistance, positioning them as a sustainable alternative to traditional metal‐based EMI shielding materials. These findings indicate that the SE of 3D‐printed materials can be substantially improved through low‐cost and straightforward PANI electrodeposition, enabling the production of customized EMI shielding materials with enhanced performance. This novel fabrication method offers promising potential for developing advanced shielding solutions in electronic devices.

## Graphical abstract

This study introduces EMI shielding bricks by 3D printing PLA composites with carbon‐based fillers such as carbon black and carbon nanotubes, followed by polyaniline coating. It examines how 0D and 1D carbon fillers influence electromagnetic interference shielding efficiency and how this can be further improved by electrodepositing with conductive polyaniline.

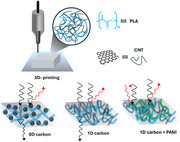

## 1 Introduction

The progress in wireless communication and microwave technology has significantly contributed to global connectivity and ease of living.^[^ ^1^ ^]^ However, this progress comes with a new problem, which is the rise in electromagnetic radiation and interference from electronic devices.^[^ ^2^ ^]^ This is more significant in X‐band frequencies (8 to 12 GHz), which are used by satellite communication, military and radar systems. Such electromagnetic interferences impact the operational integrity of electronic devices, distort information transmission and pose potential health implications for humans.^[^ ^3^, ^4^ ^]^ To overcome the challenges posed by EMI signals, the installation of an effective shielding system is highly important. Electromagnetic shields basically prevent the transmission of electric and magnetic waves between different points through the use of magnetic or conductive materials. This shielding phenomenon is the result of either reflecting the electromagnetic waves or absorbing and dissipating the radiation energy within the shielding material.^[^ ^5^ ^]^ Currently, metallic materials are most widely employed in commercial EMI shielding due to their high electrical conductivity. These materials effectively weaken electromagnetic waves by prominent skin effects, which allows the waves to penetrate only a small distance into the surface of the metal.^[^ ^6^, ^7^ ^]^ However, despite their excellent electrical conductivity, these materials are hindered by high density, restricted flexibility, and inadequate corrosion resistance, thereby limiting their broader adoption in the modern electronics industry.^[^ ^8^, ^9^ ^]^ Additionally, metallic materials usually have a reflection as a primary shielding method. However, this can lead to secondary electromagnetic wave pollution due to the re‐entry of EMI waves into the surroundings.^[^ ^10^ ^]^ Consequently, much effort has been made to develop novel materials specifically intended for high‐performance EMI shielding.^[^ ^11^, ^12^, ^13^ ^]^

Recently, 3D printing technology has revolutionized the manufacturing industry.^[^ ^14^, ^15^, ^16^ ^]^ This layer‐by‐layer printing approach permits the production of complex designs that are challenging or impossible geometries that are unachievable by traditional manufacturing methods.^[^ ^17^ ^]^ The scalability of 3D printing for industrial applications is also becoming increasingly feasible due to advances in continuous printing systems, multi‐nozzle setups, and automated post‐processing.^[^ ^18^, ^19^, ^20^ ^]^ Industries such as aerospace, automotive, and construction are already leveraging 3D printing at a large scale, with notable examples including general electric 3D‐printed jet engine parts,^[^ ^21^ ^]^ Ford 3D‐printed automotive components,^[^ ^22^ ^]^ the first metal 3D‐ printed bridge in Amsterdam^[^ ^23^ ^]^ and the construction of 3D‐printed houses.^[^ ^24^ ^]^ Among various 3D printing techniques, fused filament fabrication (FFF) is a common 3D printing method that builds models using a thermoplastic filament such as PLA, acrylonitrile butadiene styrene (ABS), polycarbonate, polyvinyl alcohol (PVA), etc., that is extruded through a heated nozzle.^[^ ^25^, ^26^ ^]^ The typical thermoplastic filaments in the FFF process are usually electrical insulators.^[^ ^27^ ^]^ These inherent insulating properties of conventional thermoplastic filaments present a major challenge to their EMI shielding properties, which rely on conductive materials that can either reflect or absorb electromagnetic waves.^[^ ^28^, ^29^ ^]^ Lately, advancements in 3D‐printed conductive filaments have shown promising improvements. This is achieved by conductive additives such as graphene, CB, carbon fibres (CF), and CNTs mixed into the thermoplastic matrix.^[^ ^30^, ^31^ ^]^ Carbon‐based materials have been explored for EMI shielding applications due to their remarkable electrical conductivity and lightweight properties.^[^ ^32^ ^]^ Recent studies on 3D‐printed honeycomb structures using PLA with graphene nanosheets and CNTs showed an EMI SE of 53.5 dB, demonstrating the importance of pore sizes in enhancing shielding performance and balancing lightweight design.^[^ ^33^ ^]^ In another study using 3D printing multifunctional conductive hydrogels using poly(3,4‐ethylenedioxythiophene) ink, a high electrical conductivity (≈2000 S m^−1^), excellent elasticity, and biocompatibility were achieved along with an excellent EMI SE of 76.4 dB.^[^ ^30^ ^]^

This 3D printing approach has several advantages over traditional techniques, as the fabrication of plain polymer layers in chemical laboratories involves intricate chemical procedures and various reagents, making the process both complex and time‐consuming.^[^ ^34^ ^]^ Additionally, creating complex 3D structural patterns using polymers often requires expensive and specialized moulding or lithographic techniques. Integrating these carbon materials into thermoplastic polymer matrices such as PLA, ABS, or PVA can improve EMI SE.^[^ ^35^, ^36^ ^]^ Additionally, combining this with 3D printing allows the fabrication of complex and customized structures with ease. In these conductive filaments, the amount of conductive carbon material plays a substantial part in shielding. If the percentage of carbon material increases, problems such as the brittleness of the filament, clogging of filament in the printer nozzle or compromise of the structural integrity of the printed structure can arise.^[^ ^37^ ^]^ However, if the carbon content is reduced, the SE will be negatively affected. The conductivity of the carbon network can be improved by incorporating an additional conductive material that links the individual carbon clusters, such as CNTs.^[^ ^38^, ^39^, ^40^ ^]^ This incorporation improves the overall performance of the composite in applications like EMI shielding. Conductive polymers such as PANI, polythiophene, polyacetylene, and polypyrrole are attractive in this sense due to their easy processability, strong corrosion resistance, lightweight nature, and applicability in various fields, including EMI shielding. Among these, PANI is more interesting due to its high conductivity, non‐redox doping, in situ polymerisation and environmental stability.^[^ ^41^, ^42^, ^43^, ^44^ ^]^ The composition of such conducting polymers with carbon‐based materials could improve the electrical conductivity of the carbon surface, improve EMI shielding performance and maintain the optimal carbon content for smooth printability.

In this study, we investigated the EMI shielding properties of 3D‐printed carbon‐based conductive bricks. Specifically, PLA with 0D CB filler and PLA with 1D CNT filler are examined with varying thicknesses. The results demonstrated that CNT/PLA bricks exhibited a SE of 43 dB at 10 GHz, more than double the 22 dB of CB/PLA bricks. To further improve the conducting environment and achieve uniformity of the CNT/PLA matrix, PANI coating was electrodeposited onto CNT/PLA bricks (PANI@CNT/PLA), resulting in a substantial improvement in SE to 54.5 dB at 10 GHz. The brick fabrication, composition and morphological properties are analysed to unveil the role of brick matric in EMI SE. In addition to their excellent SE, these bricks offer significant advantages over traditional metal‐based EMI shielding materials, including their lightweight construction, flexibility, cost‐effectiveness, and corrosion resistance. This work presents a novel approach for fabricating EMI shielding materials using 3D printing technology, offering a customisable solution for electronic devices. This research paves insight into the way to build advanced, high‐performance EMI shielding materials suitable for various applications in the electronics industry.

## 2 Results and Discussion

**Figure 1:**
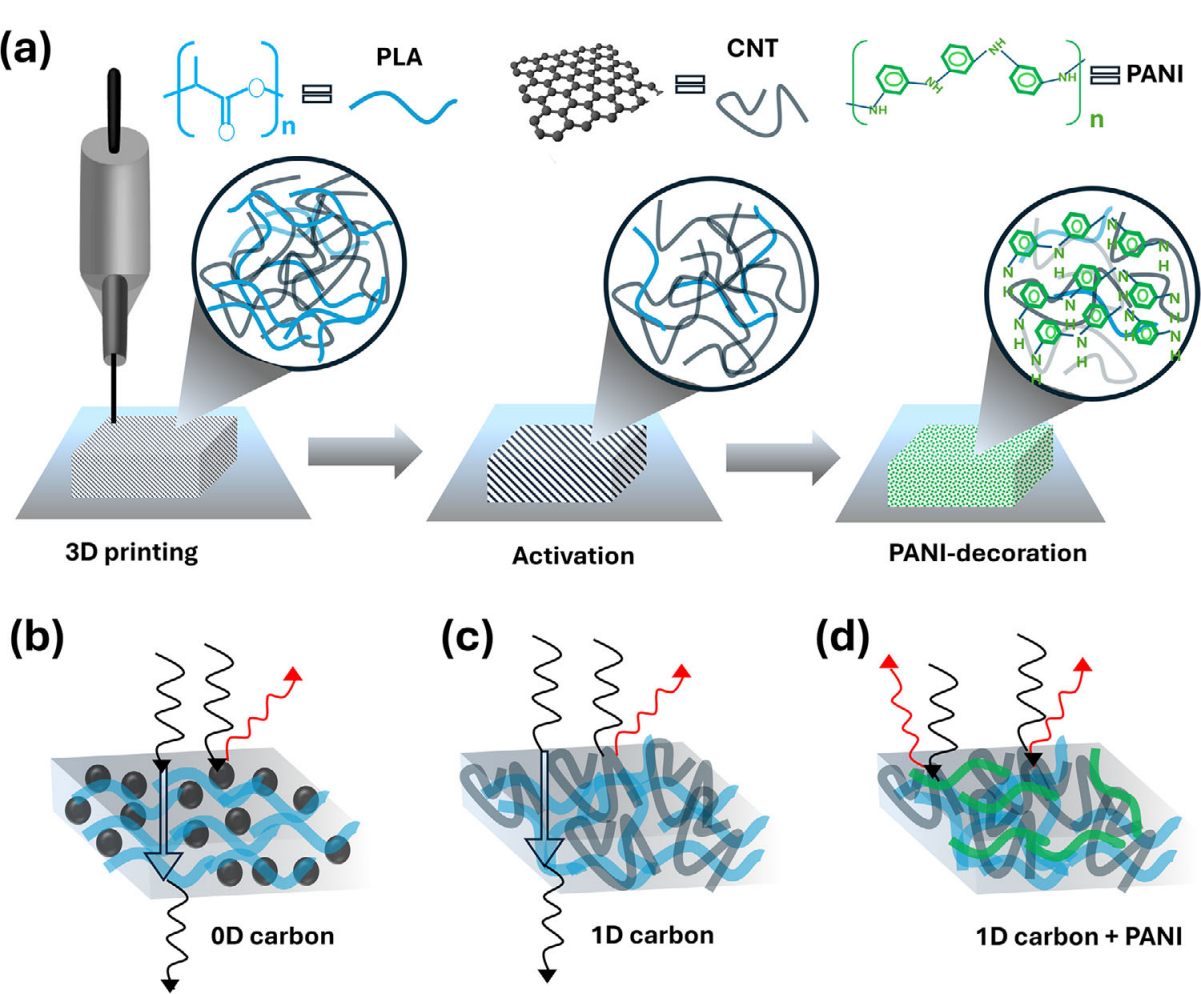
Schematic illustration of 3D‐printed EMI shielding carbon brick a) Fabrication process for PANI decorated brick; EMI shielding of b) 0D carbon (CB/PLA) c) 1D carbon (CNT/PLA) and d) PANI decorated 1D carbon.

The effectiveness of a material in EMI shielding is fundamentally related to its electrical conductivity.^[^ ^45^ ^]^ Conductive materials manage and distribute the currents induced by electromagnetic waves effectively. These induced currents generate opposing electromagnetic fields that can partially cancel out the succeeding waves, reducing the penetration of EMI through the material. Generally, higher conductivity enhances shielding performance by facilitating the attenuation of electromagnetic waves through resistive losses and reflection.^[^ ^18^, ^34^ ^]^ **Figure** **1**a illustrates the step‐by‐step procedure of the fabrication of 3D‐printed PANI‐deposited CNT/PLA EMI shielding bricks. The process starts with the 3D printing of CNT/PLA bricks using the FDM technique. Initially, the surface is covered with PLA which reduces both its surface conductivity and electrochemical activity. These bricks are then activated with sodium hydroxide solution to remove the surface PLA and expose the CNTs, as shown in Figure 1a. Subsequently, these activated bricks were then electrodeposited with PANI which covers the CNT/PLA brick surface. The shielding mechanism of CB/PLA brick is illustrated in Figure 1b. EMI shielding of CB/PLA is mainly due to the conductivity of CB particles dispersed in the PLA matrix. This moderately conductive CB network is only capable of reflecting and absorbing limited EMI waves. The EMI shielding mechanism of CNT/PLA is dissipated in Figure 1c. The higher conductivity and aspect ratio of CNTs contribute for better reflection and resistive losses of EMI waves while interacting. Furthermore, after PANI deposition, an additional conductive layer is formed on the CNT/PLA surface, which further enhances the reflection and absorption capabilities (Figure 1d). The camera image and confocal laser scanning microscopy (CLSM) of PLA, CB/PLA and CNT/PLA bricks are shown in Figure S1 (Supporting Information). Visual differences in surface texture and overall appearance of each brick can be observed. Figure S1a (Supporting Information) illustrates the surface of PLA brick with visible lines due to the layer‐by‐layer 3D printing. CLSM image and 3D height profile of PLA relatively flat surface with variations corresponding to the 3D‐printed lines (Figure S1b,c, Supporting Information). For CB/PLA, the 3D‐printed lines were more pronounced and had a slightly rougher surface texture due to the presence of CB, as shown in Figure S1d (Supporting Information). The CLSM image and height profile revealed the same with higher surface roughness compared to pure PLA (Figure S1e,f, Supporting Information). In the CNT/PLA brick, patterns were more similar to the CB/PLA brick in visual observation compared to pure PLA (Figure S1g, Supporting Information). However, the CLSM image and height profile showed rougher surface patterns compared to the CB/PLA (Figure S1h,i, Supporting Information).

**Figure 2:**
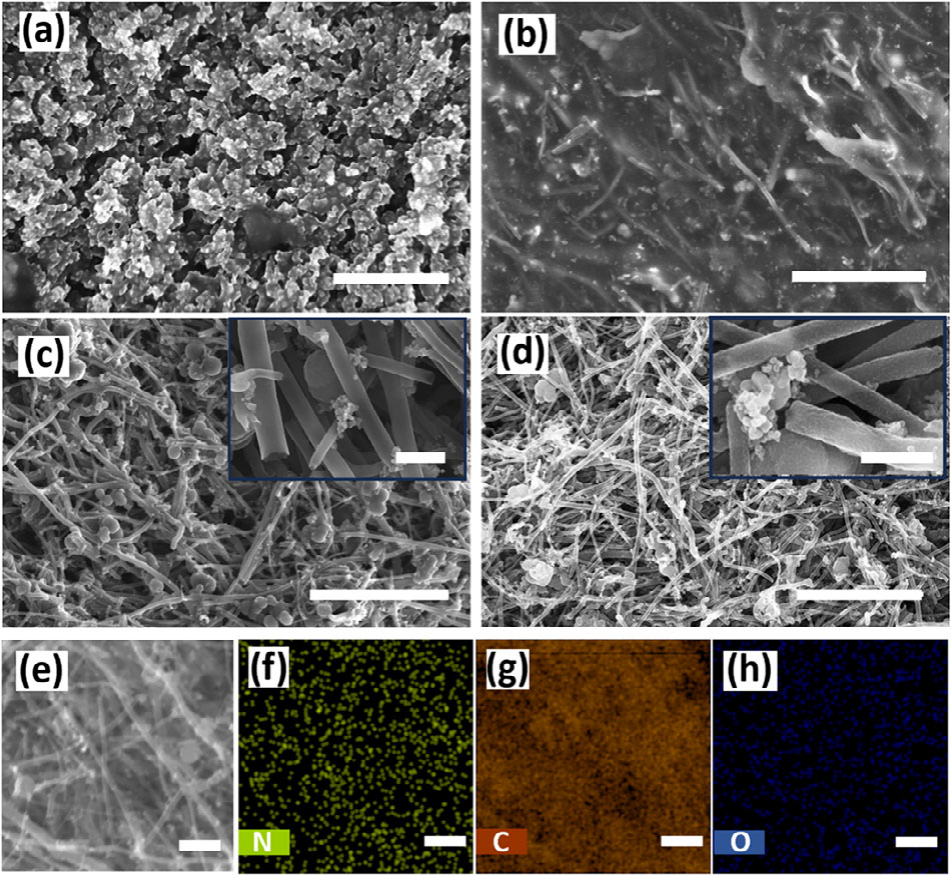
Surface morphology; SEM image of a) CB/PLA, b) CNT/PLA, c) activated CNT/PLA, and d) PANI@CNT/PLA. e) EDX mapping area and f,h) elemental mapping of N, C and O, respectively, of PANI. Scale (a–d) = 4 µm, inset image (c,d) = 300 nm, (e,f) = 1 µm.

Surface morphology studies were conducted to understand the structural and compositional changes on the brick surfaces after the activation and electrodeposition process. Scanning electron microscopy (SEM) images of bricks were taken to study the surface morphology of the bricks (**Figure** **2**). Interestingly, the CB/PLA brick surface shows CB particles covered with PLA‐forming flakes‐like structures (Figure 2a). For CNT/PLA brick, the CNTs are completely covered with PLA (Figure 2b). After the activation, the surface PLA was removed well from the surface, exposing the CNTs, which can be observed in the SEM image (Figure 2c). However, some residual PLA clusters were still observed on the surface, indicating that the activation process, while effective, did not entirely remove the polymer matrix. In PANI@CNT/PLA bricks, the coaxial deposition of PANI was observed to adhere to CNTs (Figure 2d). The elemental composition of the PANI@CNT/PLA is validated by Energy‐Dispersive X‐ray Spectroscopy (EDX) mapping, as given in Figure 2f–h. Based on the EDX spectra analysis, the atomic percentages N, C, O, Ti, and Cl are found to be 3.23%, 92.17%, 0.66%, 3.42%, and 0.53%, respectively (Figure S2, Supporting Information). The EDX mapping shows the presence of nitrogen, which comes from the PANI chains. The Ti presence can be sourced from the inherent impurity found in the CNT/PLA filament. Furthermore, the uniform deposition of PANI on the CNT/PLA is confirmed by a large area (80 µm) EDX mapping (Figure S3, Supporting Information).

**Figure 3:**
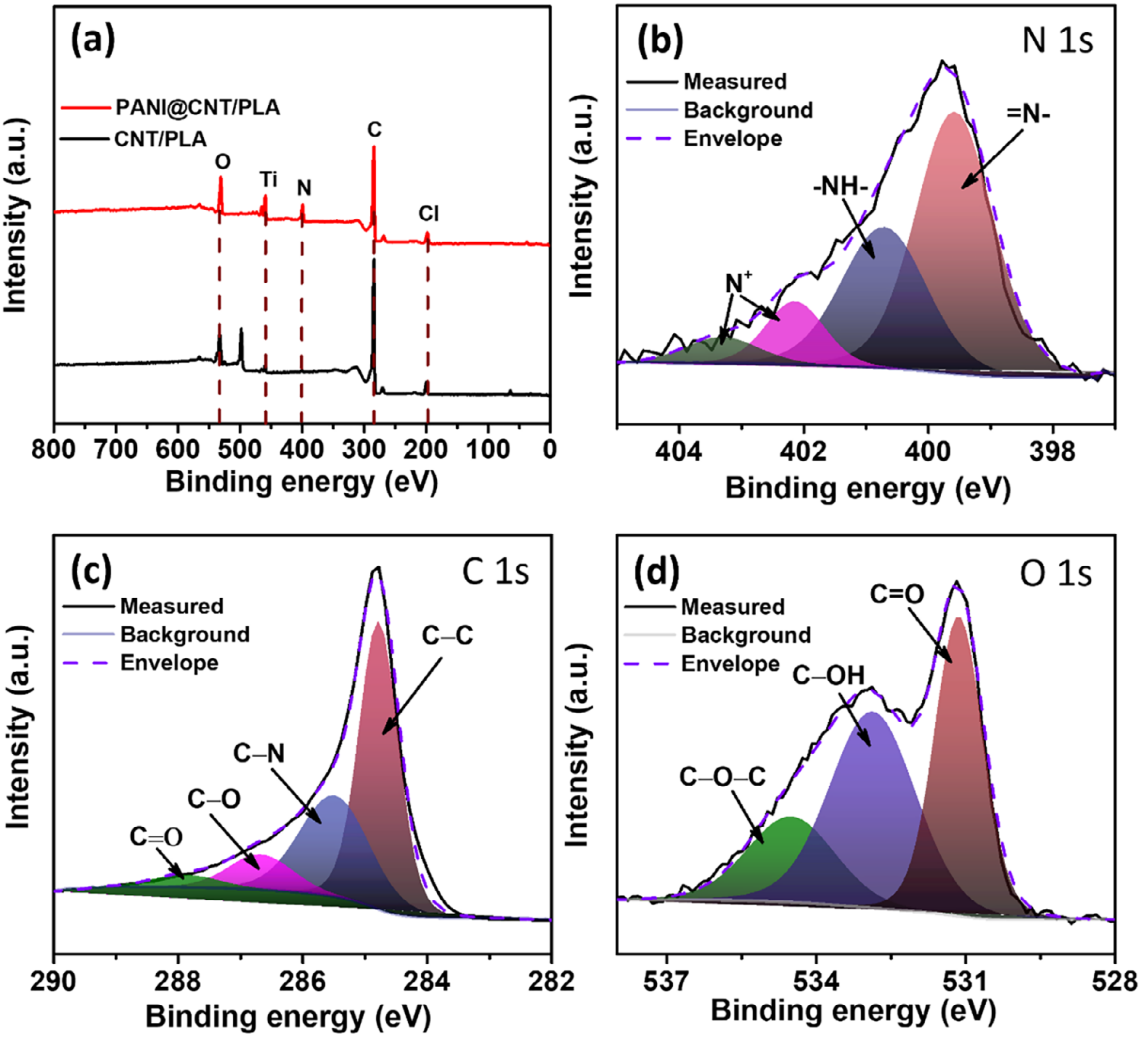
XPS analysis a) survey spectra of CNT/PLA and PANI@CNT/PLA; high‐resolution core level spectra of b) N 1s, c) C 1s, and d) O 1s.

X‐ray photoelectron spectroscopy (XPS) analysis of the bricks was carried out to study the bonding environment and elemental composition. The survey spectrum of CNT/PLA and PANI@CNT/PLA can be observed in **Figure** **3**a. From the survey spectra, the presence of N, C, Ti, Cl and O was observed. The presence of Ti can be attributed to the inherent TiO_2_ impurity in commercial 3D‐printed carbon filament. From quantitative analysis on XPS survey spectra of PANI@CNT/PLA brick, the atomic concentrations were found to be 4.37%, 77.22%, 1.36%, 9.30%, and 7.74% for N, C, Ti, Cl and O, respectively. The high‐resolution core level spectra are used for detailed analysis of the chemical states and bonding environments of individual elements present. For PANI‐coated CNT/PLA, an evident N peak was observed, confirming the deposition of PANI. The N 1s spectra of PANI@CNT/PLA can be resolved into four peaks associated with quinoid imine, benzenoid imine, positively charged imine (bipolaron state), and protonated amine (polaron state), centred at 399.5, 400.7, 402.2, and 403.3 eV, respectively (Figure 3b).^[^ ^46^ ^]^ High‐resolution spectra of C 1s shown in Figure 3c reveal the presence of four different types of carbon functional groups, that is, nonoxygenated carbon (C─C) at 284.7 eV, oxygenated carbon (C═O) in the range 287.8 eV, oxygenated carbon (C─O) in the range 286.6 eV and nitrogenated carbon (C−N) near 285.4 eV.^[^ ^26^, ^47^ ^]^ In addition, the O 1s spectrum of PANI@CNT/PLA is shown in Figure 3d. The peak at the binding energy of 534.4 eV represents C═O. The peak at 532.8 eV is assigned to the hydroxyl (C─OH) and ether (C─O─C) group peak at 531.1 eV.^[^ ^48^, ^49^ ^]^ The electrical conductivity of PANI in the CNT/PLA bricks is also influenced by its doping state, which occurs during the electrodeposition process. The undoped PANI exists in the emeraldine base form, having alternating imine (─N═) and amine (─NH─) groups in its backbone. During the electrodeposition process, the H⁺ ions from the acidic medium protonate the imine groups, converting PANI into emeraldine salt, the doped and conductive form of PANI. This doping creates charge carriers in the form of polarons (radical cations) and bipolarons (doubly charged cations), which are delocalized along the conjugated π‐electron system of PANI, enhancing its electrical conductivity.^[^ ^50^, ^51^, ^52^ ^]^ The doping level of PANI, determined from XPS analysis, is calculated to be 14.85%, based on the contributions of polarons (5.06%) and bipolarons (9.79%) to the total nitrogen content. Furthermore, the X‐ray diffraction (XRD) patterns of the CNT/PLA and PANI@CNT/PLA were observed to change in surface crystalline structure (Figure S4, Supporting Information). After PANI deposition, new 2θ peaks were observed at 20.1°, 21.9°, 25.5°, and 31.7°, with a notable increase in the peak at 25.3°. The peak at 20.1° corresponds to the (121) plane of PANI, the peak at 21.9° is attributed to the (020) plane, the peak at 25.5° is associated with the (200) plane, and the peak at 31.7° is the reflection from the (322) plane.^[^ ^53^, ^54^, ^55^ ^]^

Carbon‐based materials have been well‐studied for their shielding properties. The structural differences of CB and CNT play a crucial role in their EMI SE properties. The electronic conductivity in CB and CNT is raised from the *sp* ^2^ hybridized carbon bonding with delocalized π electrons in conjugated planar carbon backbone belonging to graphitic and graphene‐like structures. However, the conductivity of CB is usually lower than that of CNT because of the partial presence of *sp* ^3^ hybridized non‐conjugated domains in the CB. Further, CB shows conductivity from the aggregates of CB particles together, including some of the tunnelling or defect‐induced electron transport. Unlike this, CNTs contain pure graphene‐like *sp* ^2^ backbones that have higher delocalized electrons transported along the 1D‐direction to the length of CNT. However, it has lower conductivity perpendicular to the tube. Hence, in the case of 3D‐printed bricks with an insulating PLA host, the aspect ratio of conductive domains and interconnected connectivity is an important factor which affects the shielding performance.

**Figure 4:**
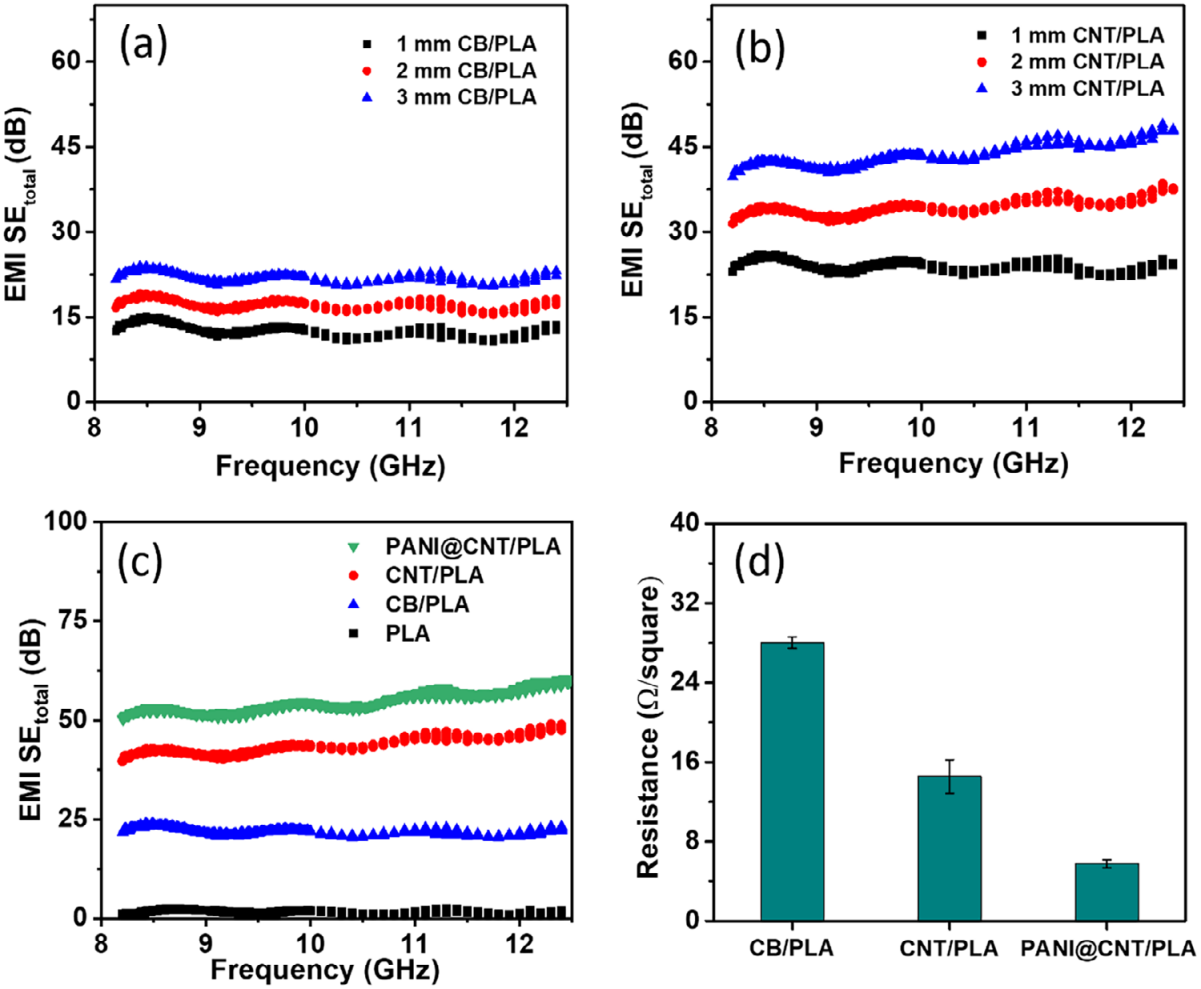
EMI SE efficiency of a) CB/PLA, b) CNT/PLA, c) comparison of 3 mm bricks PLA, CB/PLA, CNT/PLA and PANI@CNT/PLA and d) sheet resistance measurement of CB/PLA, CNT/PLA and PANI@CNT/PLA.

The effect of the carbon‐based filler structure on the EMI shielding properties of the nanocomposites is explored further. The EMI SE of the carbon‐based filler composites CB/PLA, CNT/PLA, and PANI@CNT/PLA were observed to obtain insights into the relationship between filler structure and EMI shielding performance. These composites were studied with varying thicknesses of 1, 2, and 3 mm within the frequency range of 8.2–12.4 GHz. **Figure** **4**a shows the thickness‐based comparison of CB/PLA bricks. As expected, all three samples show enhancement in EMI SE with the increase in thickness because of the combined effects of increased absorption, reflection, and attenuation. For comparison, the EMI SE of bare PLA was also studied, which shows very low shielding, as expected due to the lack of conductive pathways to absorb EMI waves (Figure S5, Supporting Information). A slight increase in EMI SE with increasing PLA thickness was observed, which can be attributed to the enhanced surface reflection (SE_R_) caused by the dielectric mismatch between PLA and air, as well as minor contributions from the layered structure and microscale voids created during the 3D printing process (Figure S6, Supporting Information). However, this reflection is insufficient for effective shielding, as PLA lacks both conductive pathways and the significant dielectric losses necessary to enhance SE_A_. The SE of CNT/PLA was observed to be higher than that of CB/PLA, indicating the influence of filler structure on SE (Figure 4b). This is due to the higher aspect ratio and better conductive network formation of CNTs, which facilitates more efficient energy dissipation through mechanisms like Joule heating and interfacial polarization, resulting in superior shielding performance.^[^ ^56^ ^]^ While CB/PLA dissipate energy primarily through conductive loss, dielectric polarization and scattering, their comparably lower conductivity and aspect ratio affect their EMI SE.

As discussed earlier, CNTs have lower conductivity perpendicular to anisotropy, while PLA also restricts the connection between CNT clusters. As observed in the spectroscopic study, PANI coating possesses polarons and bipolarons, including conjugated structures along the PANI chains that make it highly conductive. This PANI coating can lead the ways to connect the CNT domains in 3D‐network. Considering the higher EMI SE of CNT/PLA, these bricks were selected for PANI deposition. Before PANI deposition, CNT/PLA bricks were activated in the NaOH to remove partial surface PLA to expose the CNT directly to the electrolyte for efficient electrodeposition. PLA removal is begun by chemically induced hydrolysis and increases over the time of activation. The effect of activation time on SE was observed, as shown in Figure S7 (Supporting Information). The activation process facilitates better interfacial contact between the surface CNTs, as insulating PLA has decreased in the matrix. Hence, improved contacts between the CNTs enhance the electron transport pathways, thereby increasing the electronic conductivity and EMI shielding performance of the bricks. Further, it can be observed that SE performance decreased systematically from 2 to 8 h activation time, which may be assigned to the loss of active CNT content along with PLA, which is a decrease in brick thickness Therefore, PANI deposition was performed on the 8 h activated sample, which exhibited the lowest PLA content and optimal conductivity for subsequent deposition.

The EMI SE comparison of 20 cycles of PANI‐deposited CNT/PLA, CB/PLA, and CNT/PLA is shown in Figure 4c. From Figure 4c, it can be observed that the pure PLA possess minimal EMI SE due to their inability to interact with electromagnetic waves. However, the incorporation of CB improved the EMI SE moderately due to the reflection and absorption of electromagnetic waves from the conductive CB particles. Interestingly, the incorporation of CNT with PLA approximately doubled the EMI SE compared to that of CB/PLA. This can be attributed to the higher resistive losses and reflection mechanisms in CNT due to their 1D conductive structure. The PANI‐deposited CNT/PLA demonstrated the highest EMI SE, further boosting the shielding capabilities of CNT/PLA. This is greatly attributed to the delocalized π‐electrons along its conjugated polymer backbone. When electromagnetic waves strike these polymer chains, the energy is absorbed by the polymer and causes electron mobility and polarisation, which results in the conversion of energy into heat. Additionally, PANI also improves interfacial polarisation when deposited on CNT/PLA bricks. The synergic effect of the high aspect ratio of CNT and conductive networks of PANI enhances the interfacial charge transfer and dispersion of electromagnetic energy more efficiently. To investigate the effect of PANI loading on EMI shielding performance, 10, 20, and 40 cycles of PANI deposition were carried out on the CNT/PLA composite. The increase in PANI loading is verified by the atomic percentage of nitrogen, as observed in the EDX spectra at different cycles (Figure S8a, Supporting Information). As the number of deposition cycles increased, an evident increase in total EMI SE was observed in the bricks (Figure S8b, Supporting Information). This trend indicates that material loading, controlled by the number of electrodeposition cycles, significantly tunes the shielding performance of the bricks. This increase in EMI SE with the number of deposition cycles can be attributed to a higher loading of PANI, which enhances conductivity and, thereby, the ability to attenuate EMI waves.

The EMI SE of PLA, CB/PLA, CNT/PLA, and PANI@CNT/PLA composites were further evaluated in terms of their reflection (SE_R_) and absorption (SE_A_) (Figure S9, Supporting Information). PLA shows limited overall EMI SE, with the reflection being the major contributing factor. In the case of CB/PLA, the CB introduced moderate conductivity to the sample, allowing an improvement in EMI wave absorption through resistive losses. In CNT/PLA, due to the presence of CNT with a high aspect ratio and conductive 1D structure, more effective absorption of EMI waves is observed due to the improved resistive losses and better interaction with the EMI waves. The PANI@CNT/PLA composite exhibited noticeable absorption, as the PANI coating on CNTs significantly improved the conductivity and introduced delocalized charge carriers (polarons and bipolarons), which enhanced the absorption of EMI waves. This increased charge delocalization, combined with the conductive CNT network, resulted in superior dissipation of EMI energy.

The measurement of electrical conductivity can give a better understanding of the EMI SE of the materials. In this context, the electrical sheet resistance of the bricks was measured using the four‐probe method for CB/PLA, CNT/PLA and PANI@CNT/PLA, as shown in Figure 4d. CB/PLA demonstrated a sheet resistance value of 28 Ω/square. This comparably higher sheet resistance points to the fewer conductive pathways among the aggregated CB particles that limit its ability to dissipate electromagnetic energy efficiently. Whereas CNT/PLA and PANI@CNT/PLA demonstrated a sheet resistance of 15 and 7 Ω/square, respectively. The lower sheet resistance of CNT/PLA compared to the CB/PLA reflects its better conductivity networks that can effectively dissipate the EMI signals through Joule heating and interfacial polarization. The lowest sheet resistance of PANI@CNT/PLA is due to the additional conductive PANI layer. This uniform conductive layer improved the ability of the material to absorb and reflect EMI signals effectively. This is also observed in the higher EMI SE of the PANI@CNT/PLA compared to CB/PLA and CNT/PLA.

Compression and tensile tests were carried out to understand the mechanical properties of the CB/PLA and CNT/PLA samples. The compression results showed similar compressive strengths of 76.4 MPa for CB/PLA and 78.0 MPa for CNT/PLA, indicating that both fillers enhance the resistance to compressive forces (Figure S10a,b,c, Supporting Information). However, the tensile strength of CNT/PLA (2003.8 MPa) was significantly higher than CB/PLA (1140.2 MPa), attributed to the superior reinforcing ability of CNTs, which form stronger bonds within the PLA matrix (Figure S10d,e,f, Supporting Information). These results show the better mechanical performance of CNT over CB in PLA composites. Additionally, a thin film was 3D‐printed to demonstrate the flexibility of CNT/PLA. In Figure S11 (Supporting Information), images of the 3D‐printed film are shown bending at different angles to highlight its flexible structure without any deformation. However, the EMI SE of the film was relatively low (Figure S12, Supporting Information). Furthermore, a comparison study of EMI SE of PANI@CNT/PLA was made against other recently reported 3D‐printed composites (Table S1, Supporting Information). This shows the superior or comparable performance of PANI@CNT/PLA EMI shielding with respect to the reported progress of 3D‐printed EMI shielding materials. The present study indicates that the aspect ratio engineering of 3D‐printed conductive materials can be improved by introducing electrochemical methods that are effective in improving the conductivity and, hence, the EMI SE performance. To further emphasize the advantages of the PANI@CNT/PLA brick, a comparison with traditional EMI shielding metals (copper and aluminium) was conducted (Table S2, Supporting Information). The properties compared include density, EMI SE, density, corrosion resistance, flexibility, and fabrication cost. The lower density of PANI@CNT/PLA (1.04 g cm^−3^) makes it ideal for weight‐sensitive applications such as aerospace and portable devices. It also offers excellent corrosion resistance and good flexibility, unlike copper and aluminium, which are rigid and prone to corrosion. Moreover, the fabrication cost of PANI@CNT/PLA bricks is low due to the use of 3D printing and conductive polymer composites, making it more economically viable for scalable production.

## 3 Conclusion

This study demonstrates a scalable fabrication method for EMI shielding bricks using 3D‐printing technology. Initially, we investigated the effectiveness of 3D‐printed bricks containing two different carbon fillers, CNTs and CB, in shielding X‐band EMI signals. Our results indicated that bricks with CNT fillers exhibite enhanced EMI SE of 43 dB at 10 GHz, significantly outperforming CB‐filled bricks, which exhibited an SE of 22 dB at the same frequency. To further enhance the shielding performance, a conductive PANI coating was electrodeposited onto the CNT‐filled bricks, resulting in an improved SE of 54.5 dB at 10 GHz. Four probe measurements revealed differences in sheet resistance among the bricks, with the PANI‐coated CNT bricks showing the lowest sheet resistance, supporting their higher SE performance. Finally, we fabricated lightweight and efficient 3D‐printed bricks with significant EMI shielding applications. This study provides an important framework for developing 3D‐printed EMI shielding components and expands the range of 3D printing technology applications in the EMI shielding domain.

## 4 Experimental Section

### Materials

All materials used were of analytical grades, such as Aniline, obtained from Sigma–Aldrich, Germany. Sodium hydroxide (NaOH) was acquired from Penta Chemicals, Czech Republic. The conductive CNT/PLA filament (Blackmagic) was acquired from Graphene Laboratories Inc., New York, USA. CB/PLA filament (Protopasta) was acquired from ProtoPlant in British Columbia. PLA filaments were obtained from Materialpro3D.cz, Czech Republic. All experiments were conducted using deionized water with a resistance of 18 MΩ.

### 3D Printing Parameters

The bricks at different thickness were initially designed using Autodesk Fusion 360, an open‐source software. The sample was designed in a rectangular shape; it consisted of 40 mm width and 20 mm width rectangular blocks, with 1‐, 2‐ and 3‐mm thicknesses, as shown in Figure S13 (Supporting Information). The *.stl* file from Autodesk Fusion 360 was sliced and exported to a *.gcode* file with PrusaSlicer software with a 0.2 mm detail for print setting and 100% infill. The 3D printing was done on a Prusa 3D Printer (Prusa i3 MK3S, Czech Republic). For the 3D printing, the nozzle temperature is set to 215 °C and the bed temperature to 60 °C. The printed bricks were then chemically activated by soaking in 4 m NaOH for 8 h, followed by washing with ethanol and deionized water, and then dried in an electric oven at 55 °C for 2 h.

### Mechanical Characterization

The mechanical compression test and tensile strength of the samples were measured using the universal testing machine, Z010 AllroundLine, by ZwickRoell. For compression test loading rate of 2 mm min^−1^ was used to compress the samples. The samples were printed in a cylindrical shape with 6 mm diameter and height (Figure S10a, Supporting Information). Similarly, tensile test samples were prepared in dog bone shape with a thickness of 1 mm and length of 35 mm (Figure S10d, Supporting Information). A similar loading rate of 2 mm min^−1^ was also applied for tensile strength measurement.

### PANI Deposited Sample Preparation

The electrodeposition of PANI on 3D‐printed bricks were carried out by running CV following a previously reported study.^[^ ^47^, ^48^ ^]^ In short, for the electrodeposition, 10 mm of aniline solution was prepared in 0.1 m HCl solution. The deposition was conducted by performing CV cycles in the voltage range of −0.2 to +0.8 V. The PANI deposited CNT/PLA is represented as PANI@CNT/PLA in the subsequent sections.

### Characterisation

The surface features of the 3D‐printed bricks were observed by SEM FEI Verios 460L (Thermo Fisher, Czech Republic) and EDS using LYRA3 instrument (Tescan Orsay Holding, Czech Republic). The elemental composition of the 3D‐printed bricks was studied by XPS using an AXIS Supra instrument (Kratos Analytical, Japan) with a monochromatic Al Kα (1486.7 eV) excitation source. Data analysis and spectral fitting were performed using CasaXPS software. Sheet resistance measurements were carried out using Ossila T2001A3 Four‐Point Probe. EMI shielding measurements were performed using a Rohde & Schwarz ZVA Vector40 Network Analyzer connected to a WR‐90 rectangular waveguide. The samples, in the form of rectangular bricks, were positioned between the waveguides. The S‐parameters (S11, S12, S22, S21) were recorded for each sample. EMI SE was calculated using the following equation.^[^ ^57^, ^58^, ^59^, ^60^ ^]^

(1) $$\left|S_{11}\right|linear=10^{\frac{S_{11}}{20}dB}$$

(2) $$R={\left|S_{11}\right|}^{2}={\left|S_{22}\right|}^{2}$$

(3) $$T={\left|S_{12}\right|}^{2}={\left|S_{21}\right|}^{2}$$

(4) A=1−R−T

(5) $$SE_{A}=-10log_{10}\left(\frac{1-{\left|S_{11}\right|}^{2}}{{\left|S_{12}\right|}^{2}}\right)$$

(6) $$SE_{R}=-10log_{10}\left(1-{\left|S_{11}\right|}^{2}\right)$$

(7) $$SE_{T}=SE_{R}+SE_{A}$$

where R represents the reflected power, T denotes the transmitted power, and A stands for the absorbed power. Additionally, SE_A_ refers to the absorption loss, SE_R_ to the reflection loss, and SE_T_ to the total EMI SE.

## Conflict of Interest

The authors declare no conflict of interest.

## Author Contributions

S.M. conceptualized and carried out materials synthesis and fabrication, characterizations, and analysis and wrote the first draft, K.K. involved in methodology, preparing an original and revised draft and mentoring, S.S. aided with initial EMI setup and discussion of the experimental design. M.P. provided the research direction and supervised the project. All authors contributed to the writing.

## Supporting information



Supporting Information

## Data Availability

The data that support the findings of this study are available from the corresponding author upon reasonable request.
