# Supplementary material for: 3D‐Printed Nanocarbon Polymer Conductive Structures for Electromagnetic Interference Shielding
Source: Small Methods. 2025 Mar 16;9(7):2401822. doi: 10.1002/smtd.202401822 (PMC12285637; doi:10.1002/smtd.202401822)
Supplement: Supplementary file 1 — Supporting Information [file SMTD-9-2401822-s001.docx]

3D-printed Nanocarbon Polymer Conductive Structures for Electromagnetic Interference Shielding

*Shidhin Mappoli^a^, Keval Sonigara^a^, Suvani Subhadarshini^a^ and Martin Pumera^a,b,c,d*^*

^a^Future Energy and Innovation Laboratory, Central European Institute of Technology, Brno University of Technology, Purkynova 123, Brno 61200, Czech Republic

^b^Department of Chemical and Biomolecular Engineering, Yonsei University, 50 Yonsei-ro, Seodaemun-gu, Seoul 03722, Republic of Korea

^c^ Advanced Nanorobots & Multiscale Robotics Laboratory, Faculty of Electrical Engineering and Computer Science, VSB - Technical University of Ostrava, 17. listopadu 2172/15, 70800 Ostrava, Czech Republic

^d^Department of Medical Research, China Medical University Hospital, China Medical University, No. 91 Hsueh-Shih Road, Taichung, Taiwan

**
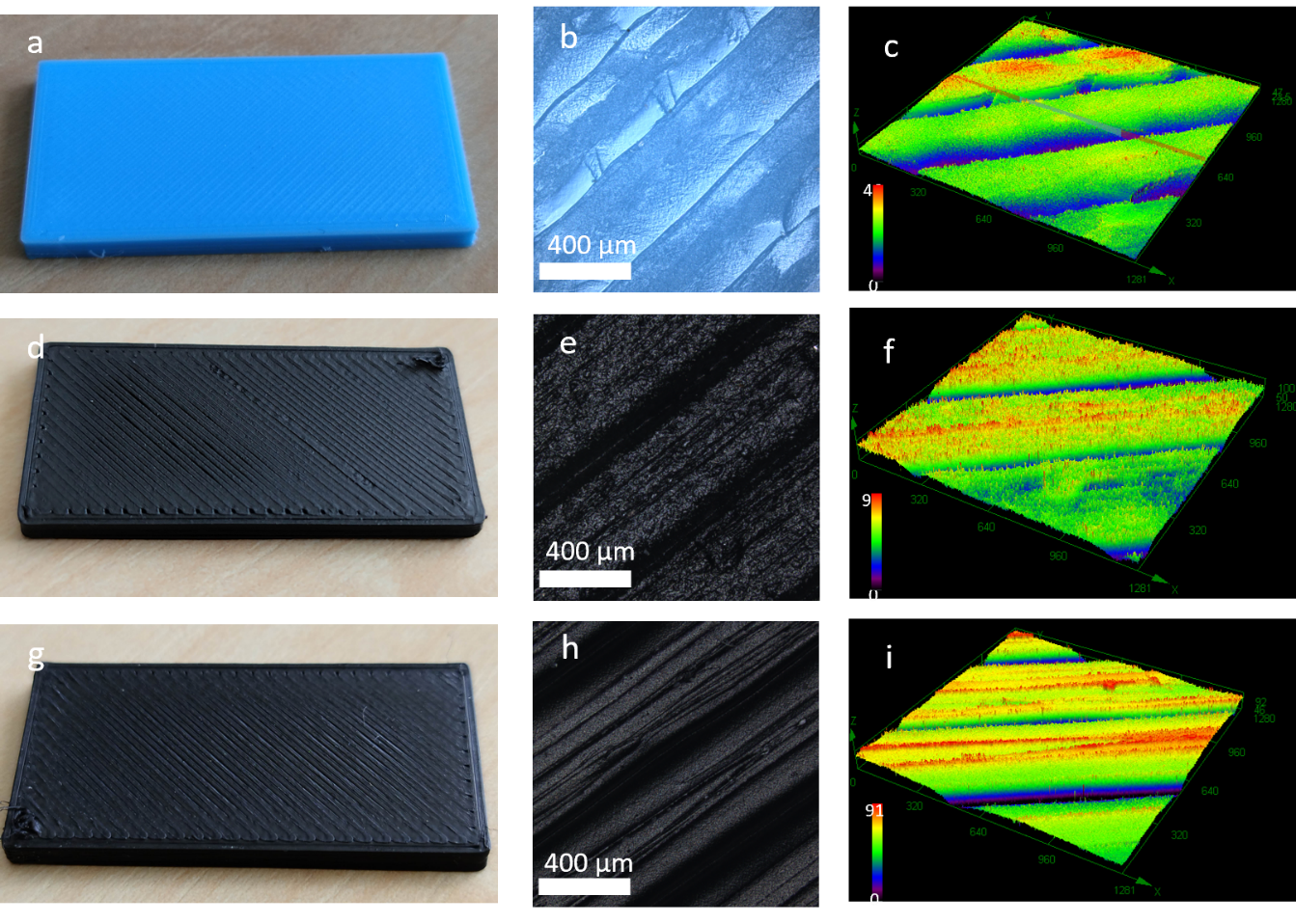
**

**Figure S1.** Surface morphology; digital camera image of a) PLA d) PLA/CD g) PLA/CNT; magnified surface image of b) PLA e) PLA/CD h) PLA/CNT; 3D surface Height profile of c) PLA f) PLA/CD i) PLA/CNT.

**
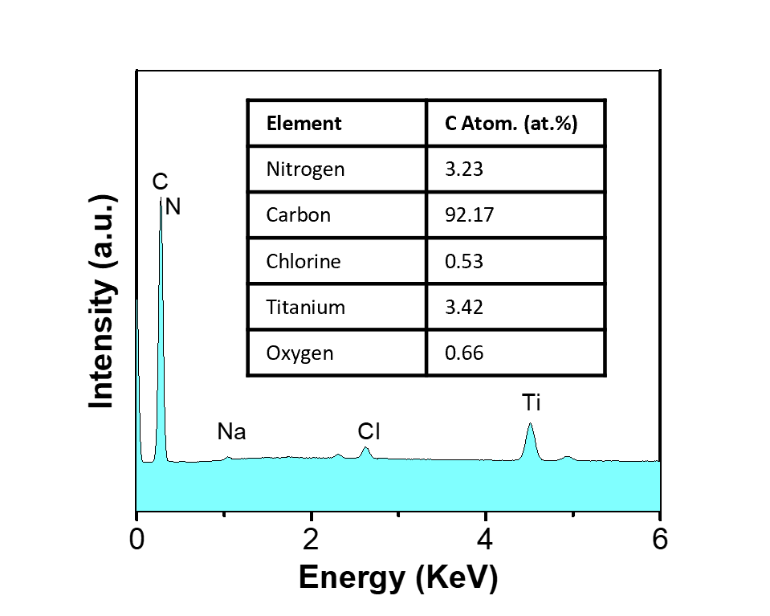
**

**Figure S2.** Scanning electron microscopy-Energy-dispersive X-ray spectra of PANI@CNT/PLA.


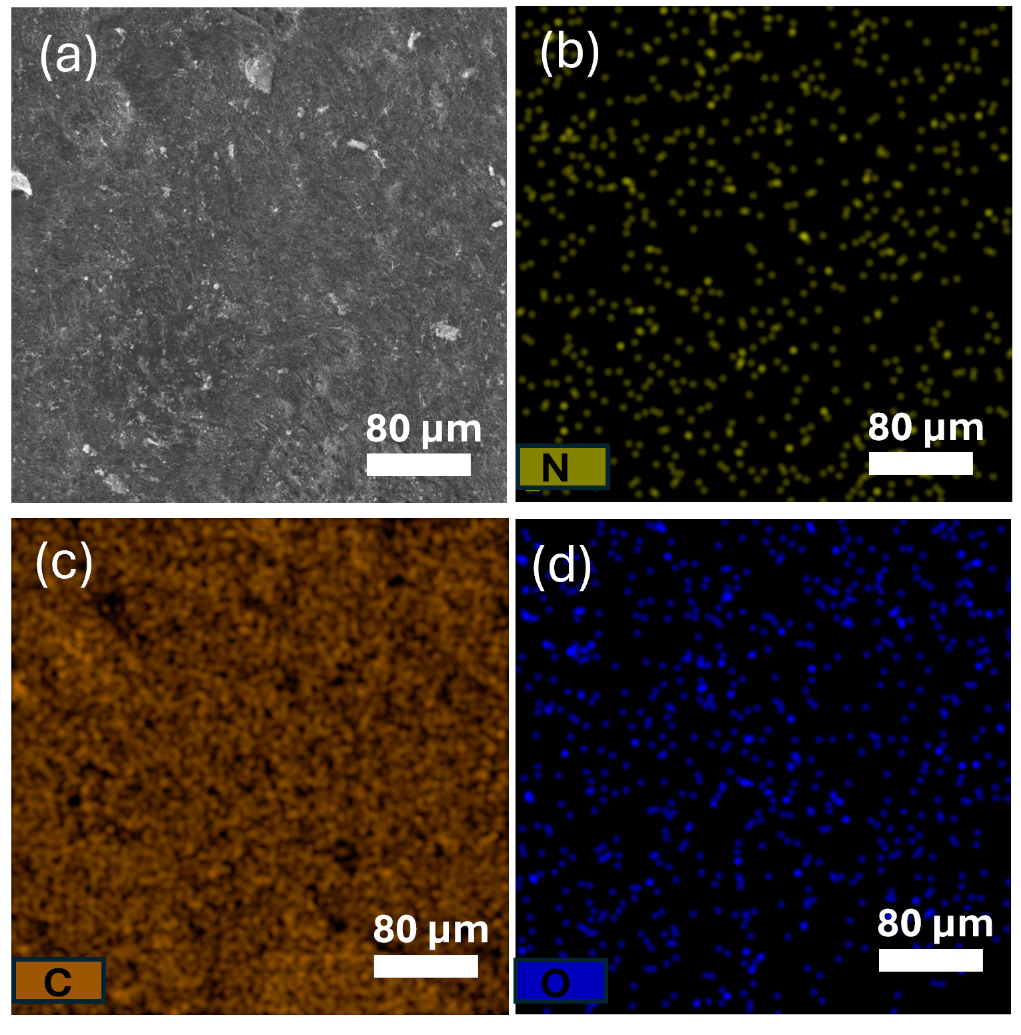


**Figure S3.** Large area EDX mapping of PANI@CNT/PLA (a) map area (b-d) elemental mapping of N, C and O, respectively.


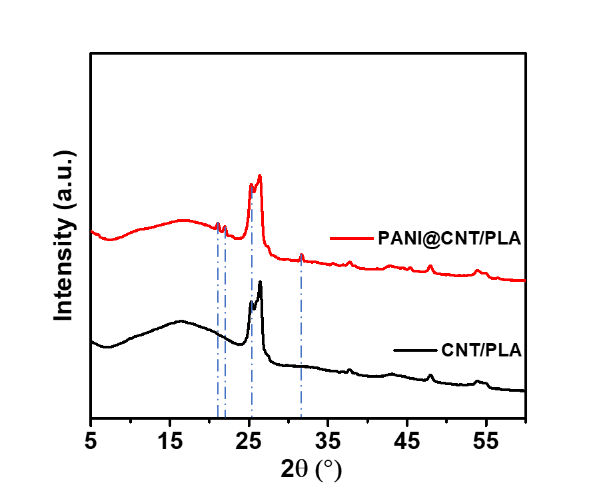


**Figure S4.** X-ray diffraction patterns of the CNT/PLA and PANI@CNT/PLA.


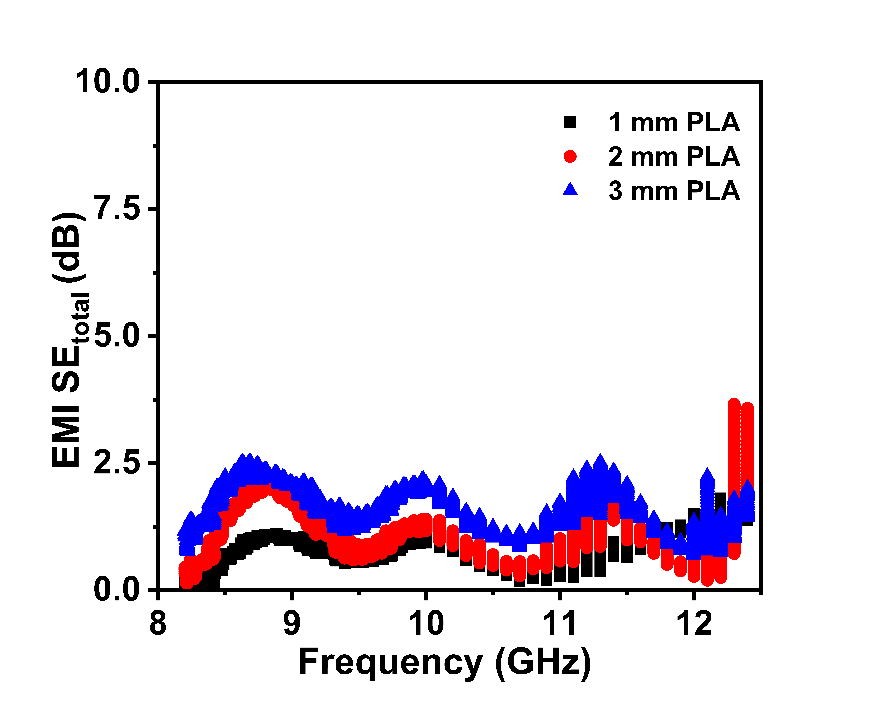


**Figure S5.** EMI SE efficiency of PLA at different thicknesses.

**
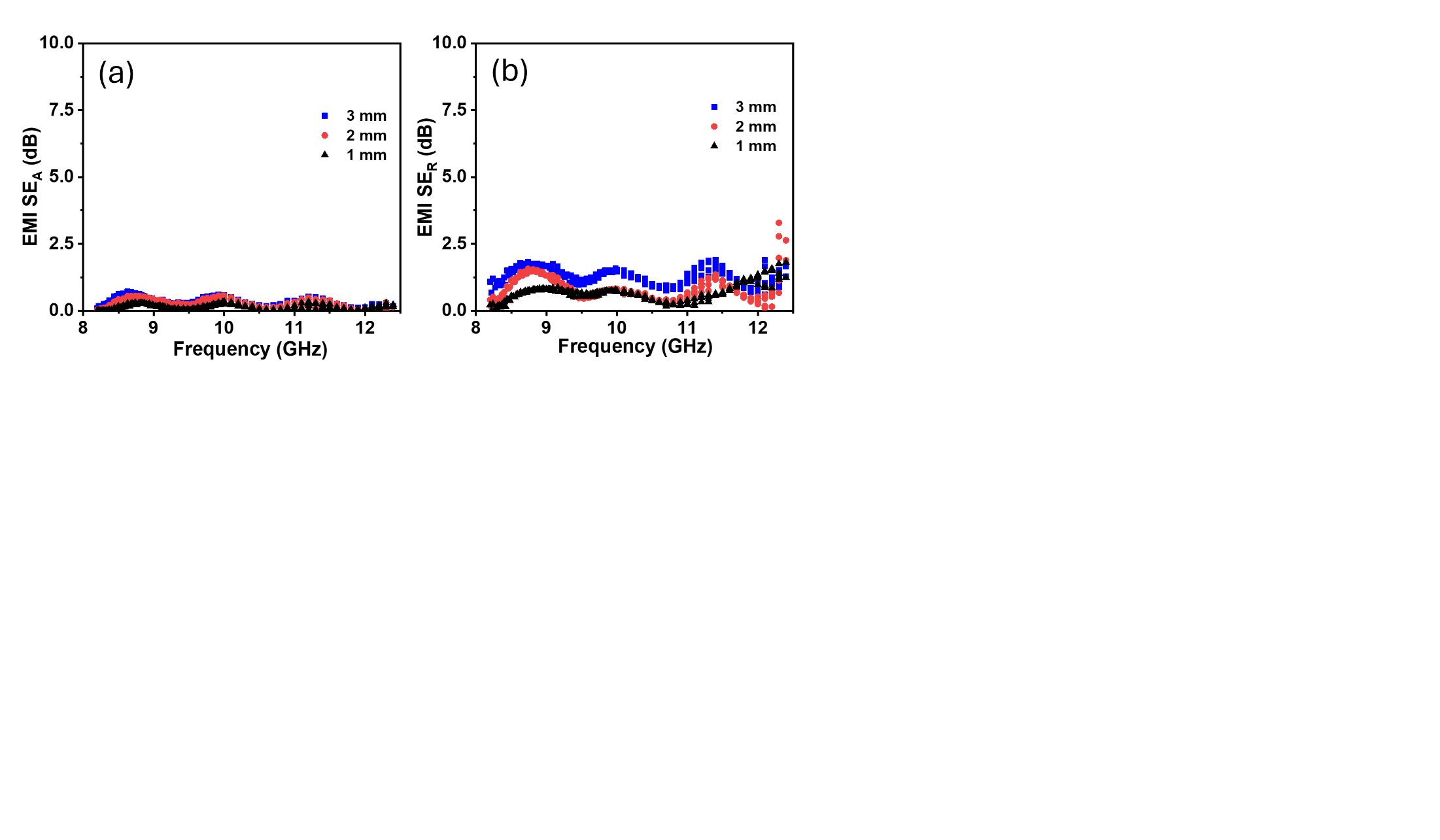
**

**Figure S6.** EMI Shielding Effectiveness due to (a) absorption and (b) reflection of PLA at 1, 2, and 3 mm.

**
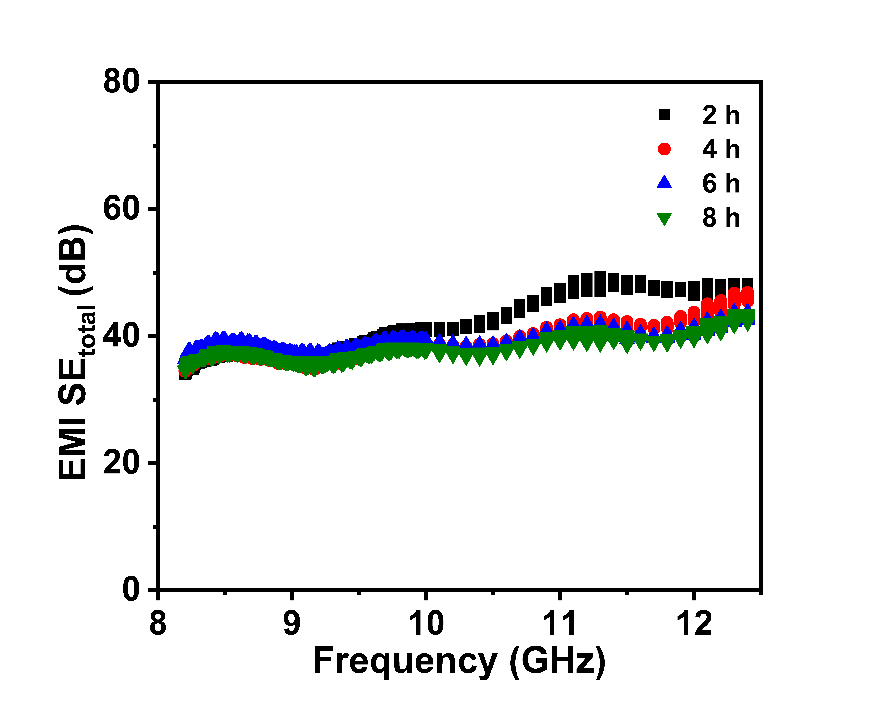
**

**Figure S7.** EMI SE efficiency of a) PLA, b) comparison of bricks after 2, 4, 6, and 8 hrs activation.


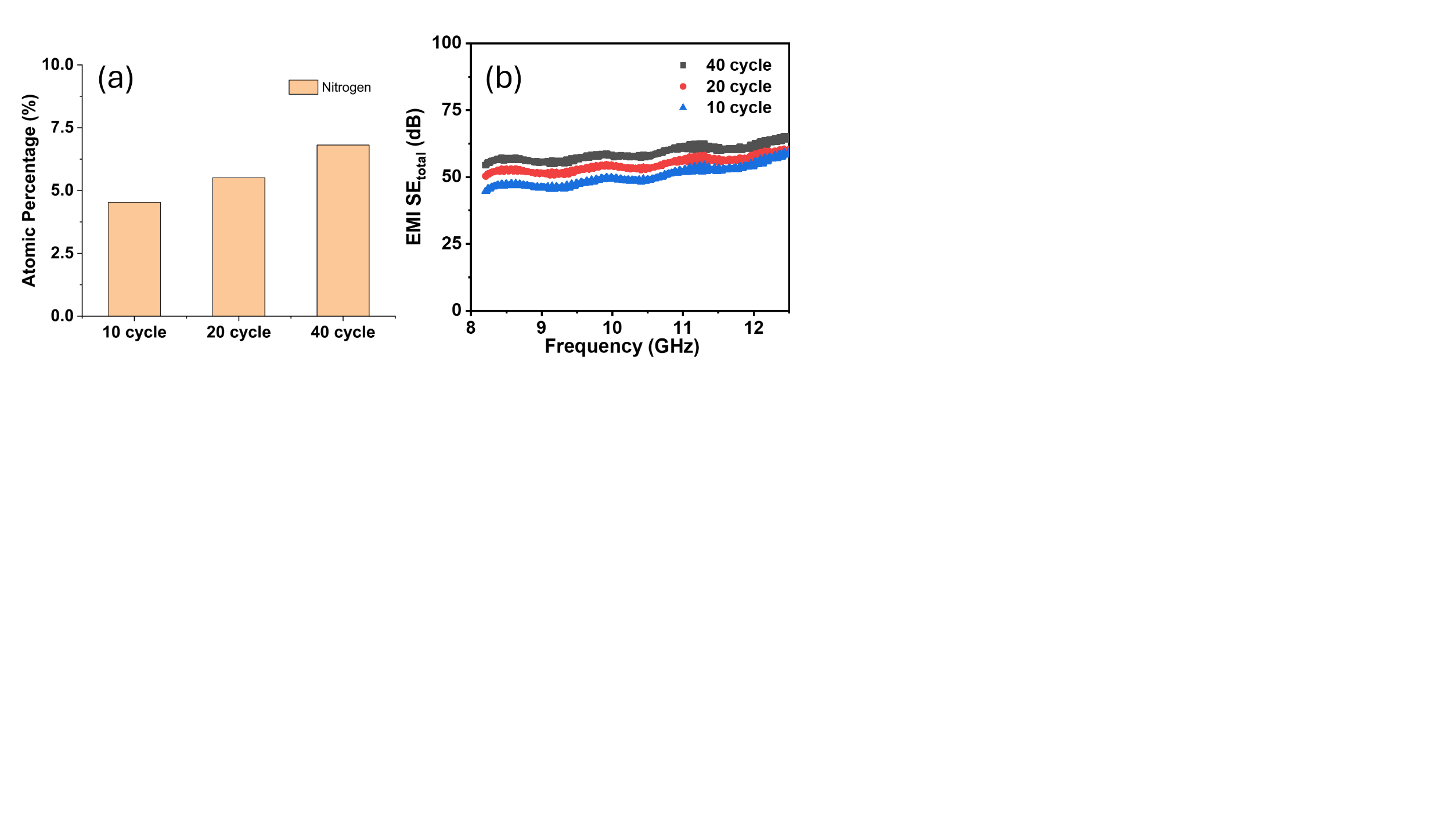


**Figure S8.** (a) EDX nitrogen atomic percentage at different electrodeposition cycles. (b) EMI SE of PANI@CNT/PLA at different electrodeposition cycles.


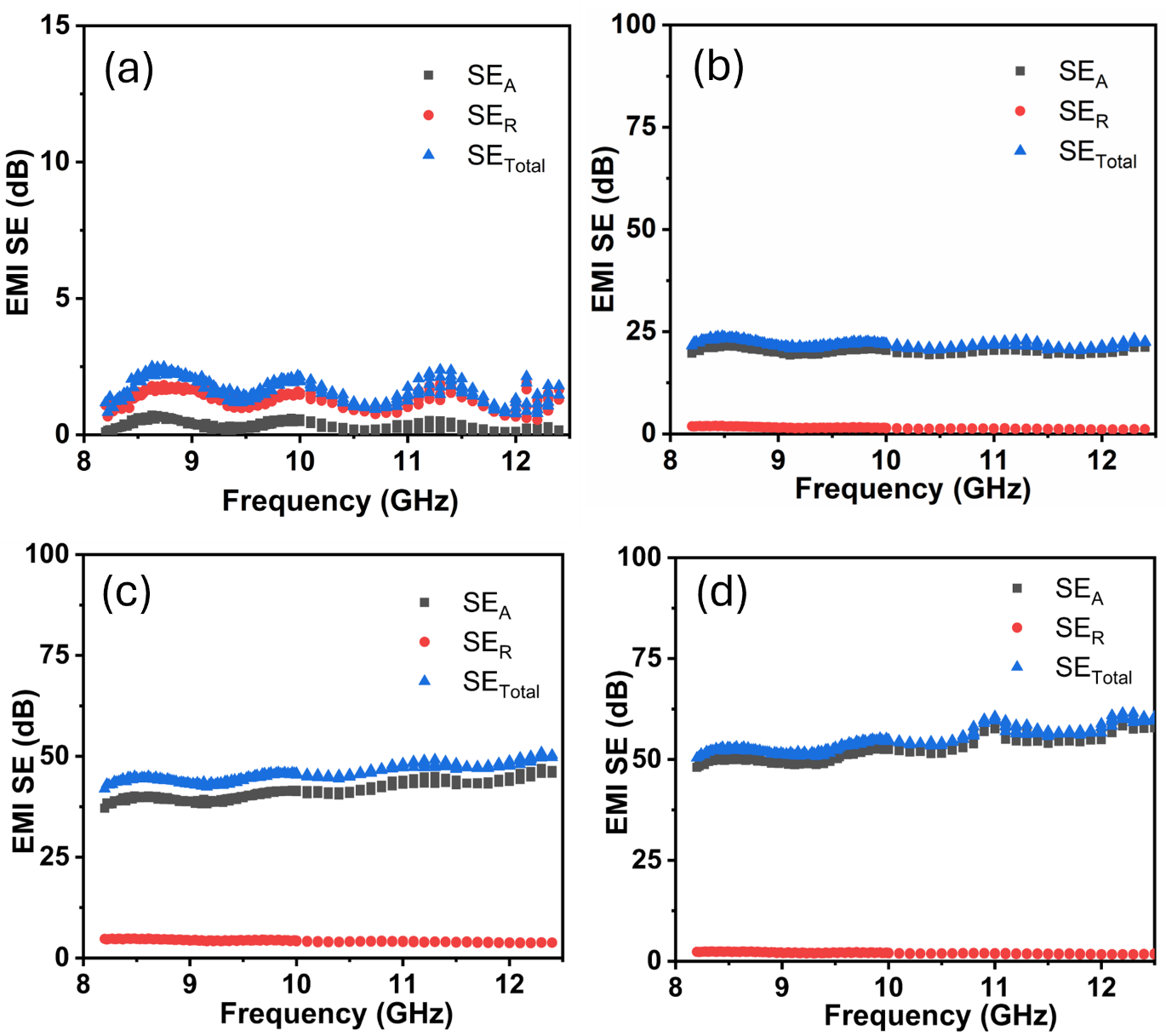


**Figure S9.** EMI SE of (a) PLA, (b) CB/PLA, (c) CNT/PLA, and (d) PANI@CNT/PLA.


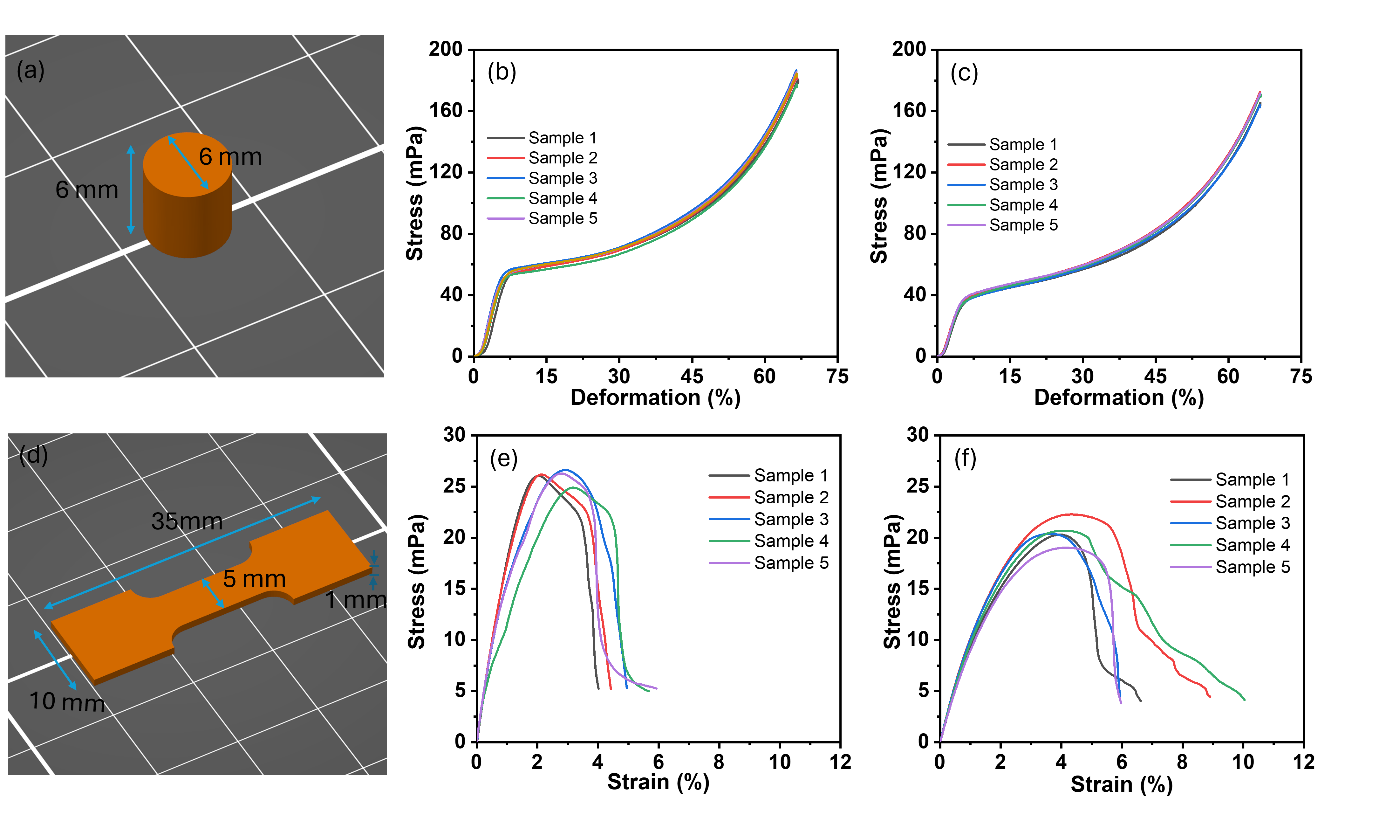


**Figure S10.** a) Model of compression test sample created in PrusaSlicer software. Mechanical compression stress vs. strain plots for b) CNT/PLA, and c) CB/PLA. d) Model of tensile test sample created in PrusaSlicer software. Mechanical tensile stress vs. strain plots for e) CNT/PLA, and f) CB/PLA.


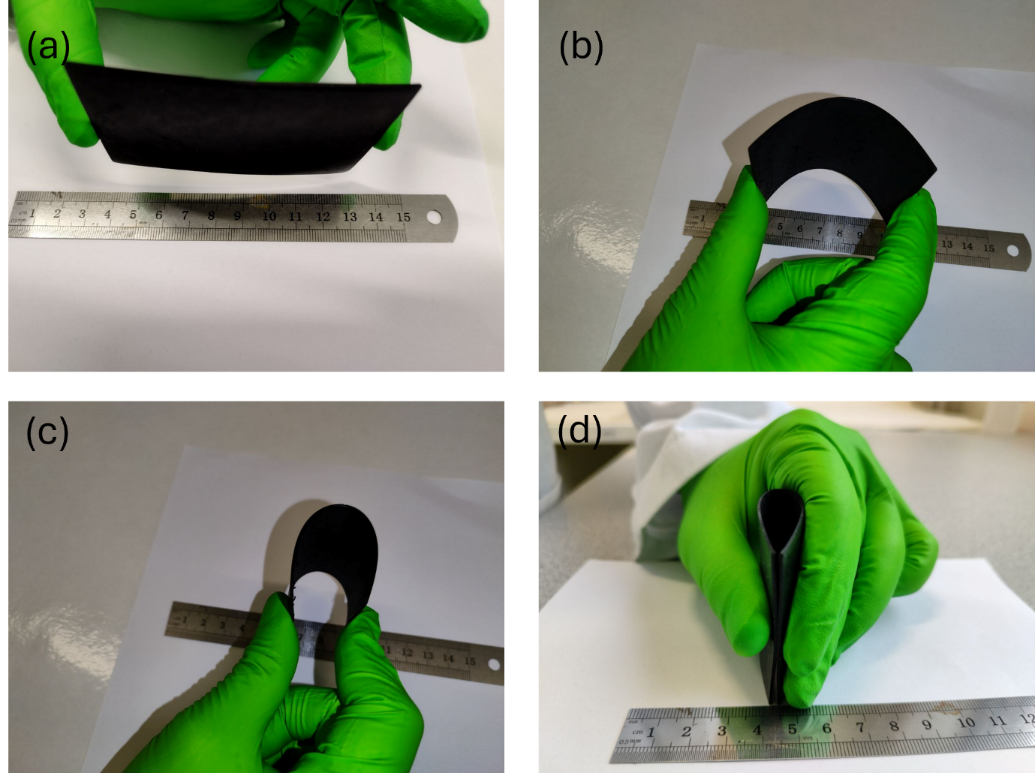


**Figure S11.** Images of the 3D printed film bending at different angles.


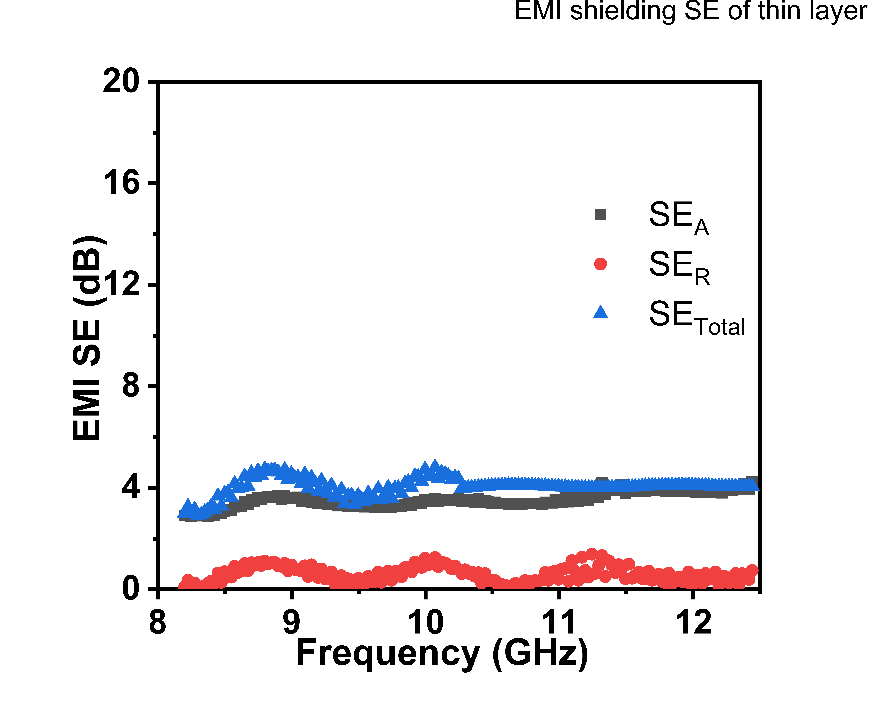


**Figure S12.** EMI SE of thin film.


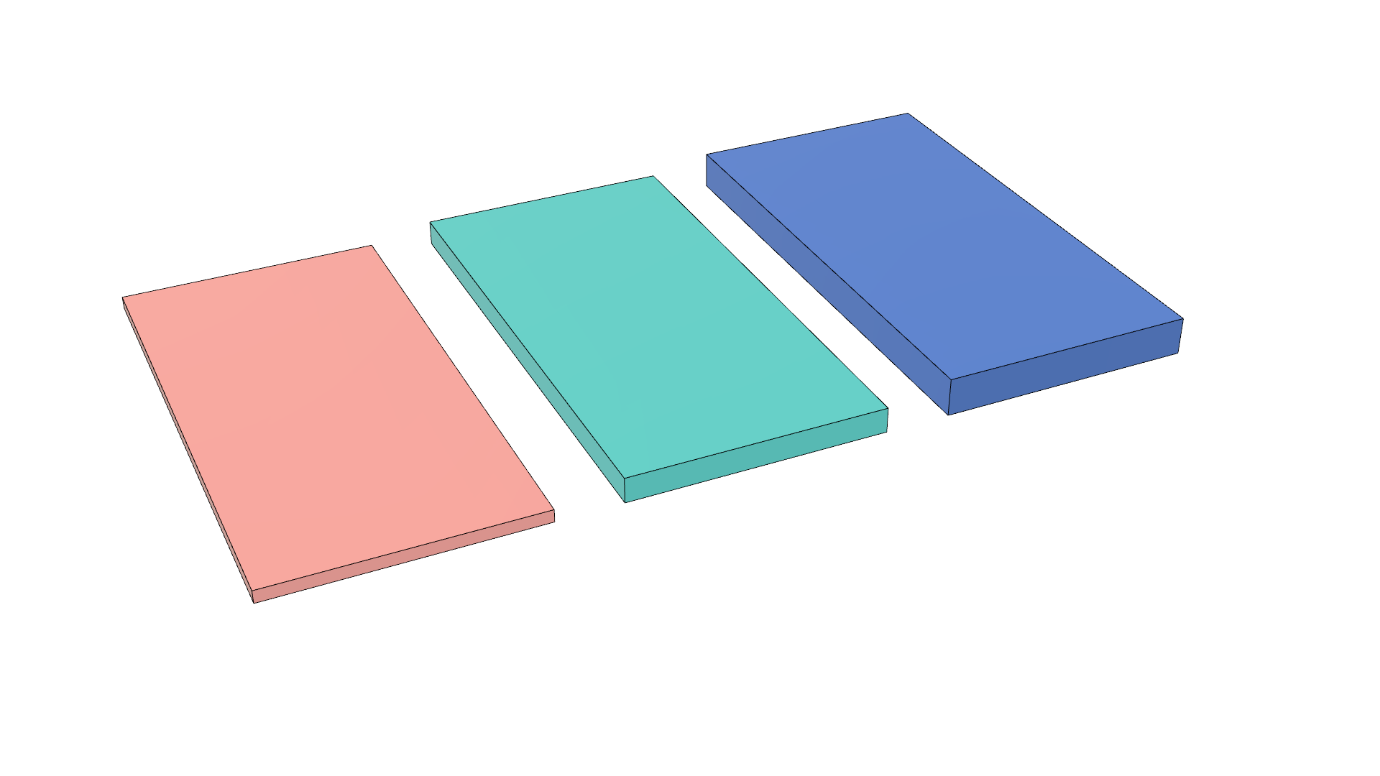


**Figure S13.** Fusion 360 images of printed bricks of different thicknesses.

**Table S1.** Comparison of EMI shielding performance of various 3D-printed materials

| **Sl.No** | **Filler** | **Polymer** | **EMI shielding effectiveness** | **Ref.** |
| --- | --- | --- | --- | --- |
| 1 | PANI@CNT | PLA | 54.7 dB (average 8.2-12.4 GHz) | This work |
| 2 | Graphene nanoplatelets | Liner low density polyethylene | ~32.4 dB | ^[1]^ |
| 3 | Carbon nanotube | ABS | -16 dB | ^[2]^ |
| 4 | Graphene nanosheets and carbon nanotubes hybrids | PLA | 35–45 dB | ^[3]^ |
| 5 | Graphene and carbon nanotube nanoparticles | Cellulose Matrix | 61.4 dB | ^[4]^ |
| 6 | Graphene Nanosheets | PLA | 35.8 dB | ^[5]^ |
| 7 | Carbon black | Epoxy Resin | ~28 dB | ^[6]^ |
| 8 | Continuous Carbon Fiber | Low-Melt Polyaryletherketone | 52.11 dB | ^[7]^ |
| 9 | Porous CNT/Ti_3_C_2_T_x_ composites | Chitosan | 26 dB | ^[8]^ |
| 10 | Carbon Fibers | Silk Fibroin | 30–31 dB | ^[9]^ |

**Table S2.** Comparison of traditional metals with PANI@PLA/CNT Bricks

| **Property** | **PANI@PLA/CNT Bricks** | **Traditional Metal Shields** (Copper, Aluminium) |
| --- | --- | --- |
| Shielding Effectiveness (SE) | 54.5 dB @ 10 GHz (3 mm thickness) | ~100 dB (0.5-1 mm) for Cu, ~80 dB (1 to 1.5 mm) for Al |
| Density | 1.04 g/cm^3^  (2.5 g @ 2 x 4 x 0.3) | 8.96 g/cm^3^ for Cu, 2.7 g/cm^3^ |
| Flexibility | Flexible at a lower thickness (~below 0.5 mm) | Rigid |
| Corrosion Resistance | High (PANI coating + polymer matrix) | Low (Requires protective coatings) |
| Environmental Impact | PLA is biodegradable, sustainable | High (non-biodegradable) |
| Fabrication Cost | Low (Single-step 3D printing) | High (Molding, assembly, welding) |
| Scalability | High (Customizable) | Medium (Complex tools needed) |

**Reference**

[1] J. Jing, Y. Xiong, S. Shi, H. Pei, Y. Chen, P. Lambin, *Compos. Sci. Technol.* **2021**, *207*, 108732.

[2] D. P. Schmitz, S. Dul, S. D. A. S. Ramoa, B. G. Soares, G. M. O. Barra, A. Pegoretti, *J. Manuf. Process.* **2021**, *65*, 12.

[3] Q. Lv, X. Tao, S. Shi, Y. Li, N. Chen, *Compos. B Eng.* **2022**, *230*, 109500.

[4] S. Shi, Y. Jiang, H. Ren, S. Deng, J. Sun, F. Cheng, J. Jing, Y. Chen, *Nanomicro Lett.* **2024**, *16*, 85.

[5] S. Shi, M. Dai, X. Tao, F. Wu, J. Sun, Y. Chen, *Chemical Engineering Journal* **2022**, *450*, 138248.

[6] Z. Wang, X. Zhang, C. Cheng, X. Song, C. Hua, L. Feng, J. Yang, J. Jiang, Y. Liu, *Compos. Struct.* **2023**, *323*, 117456.

[7] K. Abedi, S. Miri, L. Gregorash, K. Fayazbakhsh, *Addit. Manuf.* **2022**, *54*, 102733.

[8] X. Pei, G. Liu, R. Shao, R. Yu, R. Chen, D. Liu, W. Wang, C. Min, S. Liu, Z. Xu, *J Appl. Polym. Sci.* **2022**, *139*, .

[9] N. D. Sanandiya, A. R. Pai, S. Seyedin, F. Tang, S. Thomas, F. Xie, *Carbohydr. Polym.* **2024**, *337*, 122161.
